# Exploring the folding energy landscapes of heme proteins using a hybrid AWSEM-heme model

**DOI:** 10.1007/s10867-021-09596-3

**Published:** 2022-01-09

**Authors:** Xun Chen, Wei Lu, Min-Yeh Tsai, Shikai Jin, Peter G. Wolynes

**Affiliations:** 1 Center for Theoretical Biological Physics, Rice University, Houston, TX USA; 2 Department of Chemistry, Rice University, Houston, TX USA; 3 Department of Physics, Rice University, Houston, TX USA; 4 Department of Chemistry, Tamkang University, 25137 New Taipei City, Taiwan (R.O.C.); 5 Department of Biosciences, Rice University, Houston, TX USA

**Keywords:** Protein folding, Heme, Forcefield, Prediction, Nucleation mechanism

## Abstract

Heme is an active center in many proteins. Here we explore computationally the role of heme in protein folding and protein structure. We model heme proteins using a hybrid model employing the AWSEM Hamiltonian, a coarse-grained forcefield for the protein chain along with AMBER, an all-atom forcefield for the heme. We carefully designed transferable force fields that model the interactions between the protein and the heme. The types of protein–ligand interactions in the hybrid model include thioester covalent bonds, coordinated covalent bonds, hydrogen bonds, and electrostatics. We explore the influence of different types of hemes (heme b and heme c) on folding and structure prediction. Including both types of heme improves the quality of protein structure predictions. The free energy landscape shows that both types of heme can act as nucleation sites for protein folding and stabilize the protein folded state. In binding the heme, coordinated covalent bonds and thioester covalent bonds for heme c drive the heme toward the native pocket. The electrostatics also facilitates the search for the binding site.

**Supplementary Information:**

The online version contains supplementary material available at 10.1007/s10867-021-09596-3.

## Introduction

Biological physics owes a lot to the heme molecule. In the nineteenth century, Stokes, of hydrodynamics fame [1], noticed that proteins in the blood and in cells display colors which change with redox conditions. The change of color makes heme proteins ideal as experimental quantitative probes of biomolecular processes. Hemoglobin provided the first evidence of allosteric cooperativity [2], a hallmark of biological regulation. Again by monitoring color, Frauenfelder’s experiments on CO recombination inspired the notion of the complexity of protein energy landscapes, an essential cornerstone of modern biological physics [3, 4]. Indeed his observation of what has been called “polychromatic kinetics” in CO myoglobin recombination remains the clearest evidence for the necessity of using energy landscapes to describe biomolecules. Heme proteins have figured prominently in debates about folding, misleading some [5, 6] but also powering the initial explorations of how fast folding proceeds [7, 8]. In this paper, we celebrate Frauenfelder’s 100th Birthday, his work on energy landscapes and the heme molecule!

Proteins with heme groups (iron-protoporphyrin) have been studied widely for many years [9–12]. Heme proteins, including hemoglobin, myoglobin, neuroglobin, cytoglobin, and leghemoglobin, perform diverse biological functions in biosystems, including the storage and transport of small molecules, oxidation reactions, electron transport, and signal transduction [13–17].

The functional versatility of heme proteins is partly delivered by the diversity of the types of heme groups and heme-binding sites but protein dynamics modulates, the solvent accessibility of heme, the access of axial ligands to iron, as well as the distribution of hydrophilic groups around the heme important in electron transfer [13, 14, 18, 19]. For both heme b and heme c, an iron ion coordinated to a porphyrin ring binds noncovalently to the protein or to small molecules which act as additional ligands [20]. The two vinyl groups of heme b are replaced by thioester covalent bonds with cysteine side chains in heme c proteins.

While some research about heme proteins concentrates on understanding the relationship between protein structure and biological function [21–23], other studies aim to design heme proteins de novo [24]. Computational analysis has already given insight into the mechanisms of heme protein folding [25, 26]. Atomistic simulations, however, of heme proteins have allowed the exploration of how the protein energy landscape guides function. Atomistic simulations are still limited to short simulation times and experiments show that there are myraid local minima that slow dynamics during folding and function [27]. Coarse-grained simulations allow us both to survey large systems and follow their long-time motions [27].

A wide variety of protein models that depict the protein dynamics in the native state have been developed. All-atom models such as the “CHARMM” and “AMBER” forcefields have been employed to study the interactions of proteins with cofactor ligands [28–30]. We use the “AMBER” forcefield to represent the heme b and c cofactors. For the protein, we use a coarse-grained model, the AWSEM forcefield. AWSEM has already successfully surveyed many aspects of protein folding and function. It has been used not only for protein structure prediction [31], but also to study aggregation [32], and protein-DNA binding [33], and has been used for very large assemblies [34]. In these studies, coarse-grained models have already been used to explore the mechanism of heme protein folding. Interactions between the heme and protein environment have been simplified on different levels [28, 35–41]. Weinkam et al. used four pseudo atoms oriented in a square planar geometry to represent heme and a single-memory associative memory Hamiltonian for the protein-heme interaction to understand the folding mechanism of cytochrome c [42]. Ramirez et al. used 17 beads to represent heme and applied positional constraint to four heme CG beads near the pyrrolic nitrogens [43]. Harris et al. constructed the complex by Quanta/CHARMM, AMBER6, and employed the Van der Waals and the heme-cysteine linkage [44]. They have successfully explored the mechanism underlying the heme protein dynamics by simplifying heme and heme-protein interactions. We developed a knowledge-based and transferrable model for the heme-protein complex to discuss the interaction between the heme and the protein in greater detail.

In the present paper, the AWSEM-heme model is knowledge-based and transferrable. The protein is represented by the AWSEM forcefield and the heme is represented by the “AMBER” forcefield. We have considered different molecular interactions between protein and heme in this model, such as electrostatics, hydrogen bonds, and coordinate covalent bonds. Moreover, the thioester covalent bonds are included in the heme c forcefield. To understand the effects of incorporating heme in determining structure, we compared the predicted structures of heme proteins both with and without the heme molecule using AWSEM alone and the AWSEM-heme model. The heme acts to improve the quality of protein structure predictions only when the prediction of the apo form has reached sufficient accuracy. In studying the heme in the heme-binding process, we have compared several different landscapes which suggests that both the thioester covalent bonds in heme c and coordinated covalent bonds of both hemes drive the heme toward the native pocket. In the protein folding process, heme c stabilizes the folded state, which agrees with previous results with pure structure-based models [42].

## Methods

### AWSEM-heme model

We combine the coarse-grained AWSEM (Associative memory, Water-mediated, Structure and Energy Model) forcefield for proteins with the fully atomistic AMBER forcefield for heme to investigate protein-heme interactions. AWSEM is a predictive, coarse-grained protein folding model that has been used successfully to explore the folding, binding and misfolding of proteins. The forcefield is transferable and parameterized using energy landscape theory optimization from structural information on folded proteins [45]. The Hamiltonian used for the protein is made up of parts:

1 $$\begin{aligned} {H_{protein}} = {V_{backbone}} + {V_{burial}} + {V_{contact}} + {V_{HB}} + {V_{FM}} \end{aligned}$$

These terms have been comprehensively discussed by Lu et al. [46, 47]. Here, we have carried out a series of simulations using the OpenAWSEM code. The Hamiltonian used for the heme also has serveral components:

2 $$\begin{aligned} {H_{heme}} = {V_{bond}} + {V_{angle}} + {V_{dihedral}} + {V_{nonbonded}} \end{aligned}$$

These terms are exhaustively discussed by Autenrieth et al. [48]. They optimized the parameters in this forcefield using B3LYP/6-31G* calculations and compared predicted heme structures with the experimentally determined structures [48]. We used the parameters from experimentally determined structures because of the very modest differences between the purely theoretical calculations and experimentally determined structures [48]. Besides the Hamiltonian for the protein and the heme individually, we introduce knowledge-based Hamiltonians for the heme b-protein interaction as having components:

3 $$\begin{aligned} H_{heme b-protein} = {V_{Fe - cc}} + {V_{elec}} + {V_{hb-backbone}} + {V_{hb-sidechain}} + {V_{burial}} + {V_{excl}} \end{aligned}$$

The coordinated covalent bond between Fe and residues is maintained through the potential $V_{Fe-cc}$:

4 $$\begin{aligned} {V_{Fe - cc}} = \sum \limits _{i = 2}^{N - 1} {{\lambda _{Fe - cc,i}}[{e^{{{({r_i} - {r_{Fe-cc}})}^2}}}\prod \limits _j^4 {\frac{{1 + \tanh (2*(\sin ({\theta _{ij}}) - \sin ({\theta _{Fe-cc}})))}}{2}]} } \end{aligned}$$

The values of ${\lambda _{Fe-cc,i}}$, the equilibrium distance ${r_{Fe-cc}}$ and the equilibrium angle $\theta _{Fe-cc}$ are given in Table S1. N is the number of residues. The first and last residues as well as glycine are not included in this potential where $r_{i}$ is the distance between the C$\beta$ atom of residue i and the Fe atom of heme b. $\theta _{ij}$ is the angle formed the C$\beta$ atom of residue i, the Fe atom of heme b and the N atom j of heme b. The Hamiltonian has a distance dependant term ${{e^{{{({r_i} - {r_{Fe-cc}})}^2}}}}$ and an angular part ${\prod \limits _j^4 {\frac{{1 + \tanh (2*(\sin ({\theta _{ij}}) - \sin ({\theta _{Fe-cc}})))}}{2}} }$. The distance-dependent part restrains the distance between heme b and the C$\beta$ of the residue. The angular part keeps C$\beta$ atom staying directly above the Fe atom of heme b binding perpendicular to the heme b plane. $V_{elec}$ describes the electrostatic interactions between heme b and protein:

5 $$\begin{aligned}{V_{elec}} = \lambda _{elec} \sum \limits_i^N \sum \limits _j^n {\frac{charge_{i}*charge_{j}}{r_{ij}}}\mathrm{e}^{ (-{\frac{k_{screening}*{r_{ij}}}{l_{screening}} )}}\end{aligned}$$

The values of $\lambda _{elec}$, the screening length $l_{screening}$, and $k_{screening}$ are shown in Table S2. $charge_{i}$ is the charge of residue i and $charge_{j}$ is the charge of heme b atom j. The charge on each of the heme b atoms is computed by B3LYP/6-31G* from Autenrieth et al.’s work [48]. $r_{ij}$ is the distance between C$\beta$ atom of residue i and heme b atom j. The hydrogen bond interactions between heme b and the protein backbone is defined as $V_{hb-backbone}$:

6 $$\begin{aligned} {V_{hb - backbone}} = {\lambda _{hb - backbone}}\sum \limits _{i =2}^{N - 1} {\sum \limits _j^n {} {e^{ - 4{{({r_{ij}} - {r_{HB}})}^2}}}} \frac{{\tanh (4({\theta _{ij}} - {\theta _{HB}}) - 6) + 1}}{2} \end{aligned}$$

In AWSEM, each residue except glycine is described by three atoms (C$\alpha$ atom, C$\beta$atom, O atom). The coordinates of other atoms in the backbone, such as the N atom, the C atom, and the H atoms, can be determined assuming an ideal geometry. Here, the backbone’s hydrogen bond term describes the hydrogen bond formed between the N-H groups and the carboxyl groups of heme b. The values of $\lambda _{hb-backbone}$, hydrogen bond distance $r_{HB}$, and the hydrogen bond angle $\theta _{HB}$ are shown in Table S2. $r_{ij}$ is the distance between the H atom of residue j and the carboxyl O atom j of heme b. ${\theta _{ij}}$is the angle between the N atom of residue i, the H atom of residue i, and the carboxyl O atom j of heme b. $V_{hb-sidechain}$ is a hydrogen bond term that provides an interaction between protein sidechains and carboxyl groups of heme b:

7 $$\begin{aligned} {V_{hb - sidechain}} = \sum \limits _{i=2}^{N - 1} {\sum \limits _j^n {} {\lambda _{i,HB}}{e^{ - 4{{({r_{ij}} - {r_{i,HB}})}^2}}}} \end{aligned}$$

The values of $\lambda _{i,HB}$ and equilibrium distance $r_{i,HB}$ are shown in Table S3. $r_{ij}$ is the distance between C$\beta$ atom of residue i and carboxyl O atom j of heme b. The carboxyl group is hydrophilic and this prefers to interact with hydrophilic residues and water. The $V_{hb-sidechain}$ has already encoded the hydrophilic interactions between carboxyl groups and hydrophilic residues. Here we have introduced the $V_{burial}$ term to implicitly describe the hydrophilic interactions between carboxyl groups and water:

8 $$\begin{aligned} {V_{burial}} = {\lambda _{burial}}\sum \limits _i^N {\sum \limits _j^n {} } \frac{{(1 - \tanh (8({r_{ij}} - {r_{burial}})))}}{{4N}} \end{aligned}$$

The values of $\lambda _{burial}$ and the cutoff distance $r_{burial}$ are shown in Table S2. $r_{ij}$ is the distance between C$\beta$ atom and reside i and carboxyl O atom j of heme b. $V_{excl}$ is an excluded volume interaction between heme b and the protein that prevents atoms at short distances from overlapping:

9 $$\begin{aligned} {V_{excl}} = {\lambda _{excl}}\sum \limits _i^N {\sum \limits _j^n {{{({r_{ij}} - {r_{excl}})}^2}step({r_{excl}} - r)} } \end{aligned}$$

The ${\lambda _{excl}}$ and cutoff distance $r_{excl}$ are shown in Table S2. $r_{ij}$ is the distance between atom i of the protein and atom j of heme b. Heme sometimes exists as heme c in biosystems, which forms two thioester covalent bonds with protein cysteines. To describe this, we have introduced another interaction energy between heme c and protein:

10 $$\begin{aligned} {H_{heme c-protein}} = {H_{heme b-protein}} + {V_{thioester}} \end{aligned}$$

$H_{heme b-protein}$ has already been introduced above. The $V_{thioester}$ term maintains the thioester covalent bonds between heme c and the protein:

11 $$\begin{aligned} {V_{thioester}} = {\lambda _{CS}}\sum \limits _i^{{N_{cys}}} {\sum \limits _j^{{N_{thioester}}} {{e^{ - 4{{({r_{ij}} - {r_{thioester}})}^2}}}(1 - \tanh (3({r_{ij}} - {r_{thioester}})))(1 + \tanh (32({r_{ij}} - {r_{thioester}})))} } \end{aligned}$$

The values of $\lambda _{thioester}$ and the cutoff distance $r_{thioester}$ are shown in Table S2. $r_{ij}$ is the distance between C$\beta$ atom i of two cysetines separated by two residues in the protein and atoms of vinyl groups in heme c.

### Metrics of structual similairity

Two metrics were used to evaluate the accuracy of predicted structures when compared to crystal structures from different levels. From the protein level, we used $Q_{w}$ to measure the structural accuracy of the protein itself, as shown below:

12 $$\begin{aligned} {Q_w} = \frac{2}{{(N - 2)(N - 3)}}\sum \limits _{j - i > 2} {{\mathrm{e}^{\frac{{ - {{({r_{ij}} - {r_{ij}}^N)}^2}}}{{2{\sigma _{ij}}^2}}}}} \end{aligned}$$

13 $$\begin{aligned} {\sigma _{ij}} = |j - i|{^{0.15}} \end{aligned}$$

$r_{ij}$ is the distance between C$\alpha$ atom of residue i and C${\alpha }$ atom of reside j in predicted protein structure. ${r_{ij}}^N$ is the distance between corresponding atoms in experimental protein structures. $\sigma _{ij}$ is a sequence separation-dependent well width. To describe the protein–ligand geometry, the quantity $Q_{c}$ was introduced to measure whether the heme has found the experimental pocket in the predicted structure:

14 $$\begin{aligned} {Q_c} = \frac{N}{{{N_e}}} \end{aligned}$$

$N_{e}$ is the number of contacts between heme and protein in the experimentally determined structure. We counted a contact as being formed when the distance between the atom of heme and the C$\beta$ atom of protein is less than 0.65 nm. *N* is the number of contacts between the heme and the protein in the predicted structure, where the contacts should also exist in the crystal structure. The Qc value is normalized so as to vary between 0 and 1.

### Simulation details

We first set up simulations for heme b proteins and another series of simulations for heme c proteins for each one, a series using a single memory and another series that employed fragment memories. To understand the role of heme in structural prediction, we also ran protein simulations without any heme for comparison. All predicted simulations used an annealing protocol ranging down in temperature from 800K to 200K in 4 million steps (20 $\mu$s in lab time) implemented using OpenAWSEM, starting with the heme far from a disordered protein. Some hemes form two coordinated covalent bonds with protein simultaneously, but some hemes use one of their available coordinated covalent bonds to bind a small molecule such as oxygen rather than the protein. To prevent unrealistic binding, we added the same small molecule found in the crystal structure and constrained it tightly to remain near the heme. For cytochrome c (PdbID:1HRC), two sets of umbrella sampling were carried out at 300K using the $Q_{w}$ of the protein as the reaction coordinate to compute the free energy differences between the apo form and the holo form. Both sets of simulations started with the protein in an extended state and ran four million steps (20 $\mu$s in lab time), which is enough to ensure reasonable convergence of sampling.

## Results

### The incorporation of Heme b enhances the accuracy of structure prediction

**Fig. 1:**
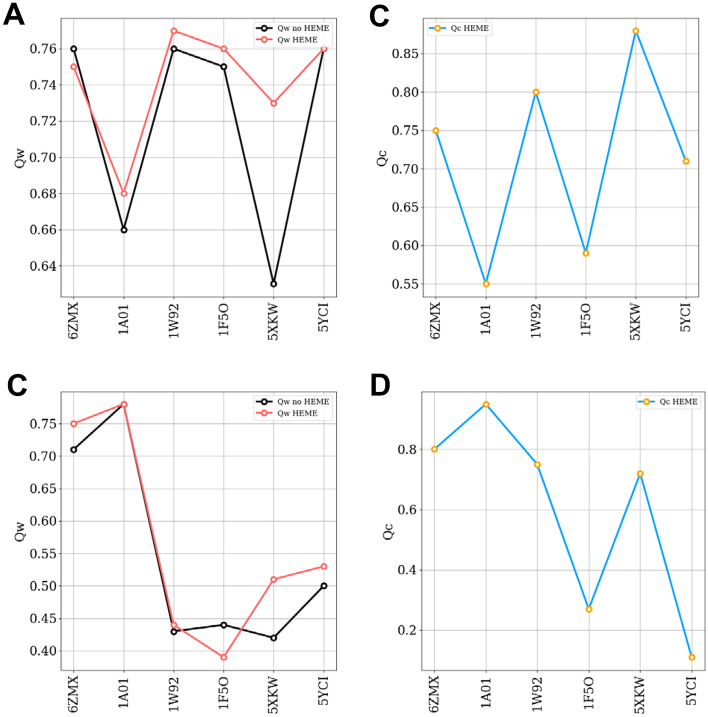
Summary of heme b protein predictions. **A** The $Q_{w}$ between best structure in prediction and experimental determined structure by different setups using single memory. Black: Apo form, Red: Holo form. **B **The $Q_{c}$ between best structures in holo form predictions using single memory and experimentally determined structures. **C** The $Q_{w}$ between best structures in predictions and experimental determined structures by different setups using fragment memory. Black: Apo form, Red: Holo form. **D** The $Q_{c}$ between best structures in holo form predictions using fragment memory and experimentally determined structures

We have summarized the results of structure prediction using a single memory in Fig. 1. These are meant to specifically test the heme protein interactions. We have compared the maximum $Q_{w}$ values achieved by the apo form (Black) and the holo form (Red) prediction runs for a particular target sequence versus its sequence length in Fig. 1(A). Also, we have plotted in Fig. 1(B) the corresponding $Q_{c}$ that measures the fraction of native contact formed in prediction run between protein and heme. AWSEM performs well using single memory: all of the $Q_{w}$ for the best structure in apo form prediction are above 0.63. This means that the protein can fold toward the native holo state without heme b. The results from the holo form predictions, however, are generally improved when compared to the results of apo form prediction runs. Most hemoglobins and neuroglobin (PDBID:1A01,1W92,6ZMX) show slight enhancement in quality comparison to the apo form prediction. With Heme, the prediction of one myoglobin (PdbID 5XKW) improved significantly over the prediction done without heme, whose $Q_{w}$ increases by 0.1. Another myoglobin holo form prediction does not improve much (PdbID:5YCI). The holo form predictions always outperform apo form predictions when the apo form predictions themselves are sufficiently accurate.

Purposefully excluding the knowledge of the crystal structures of the protein and of its homologues, fragment memories were generated by searching the database using each fragment for individual 9 residue long sequences. The results using fragment memories as local biases are shown in Fig. 1. In addition to the apo form predictions (Black), we show the maximum $Q_{w}$ values achieved by holo form (Red) versus sequence length in Fig. 1(C). We have also plotted the $Q_{c}$ values of the predicted structures with maximum $Q_{w}$ value versus sequence length in Fig. 1(D). AWSEM with fragment memories does not perform as well as AWSEM with single memory, as expected. Generally, while the holo form predictions outperform the apo form predictions, they do not improve as much as the predictions using only a single memory did. The improvement again depends on the quality of the apo form predictions, introducing the heme b can improve the quality of structure prediction only when the apo form predictions already perform well enough. The heme can find its native pocket only when the apo form prediction is accurate enough, indicating that the heme b binding accuracy depends on the quality of protein structure prediction.

### Heme b can find native pocket during protein folding

**Fig. 2:**
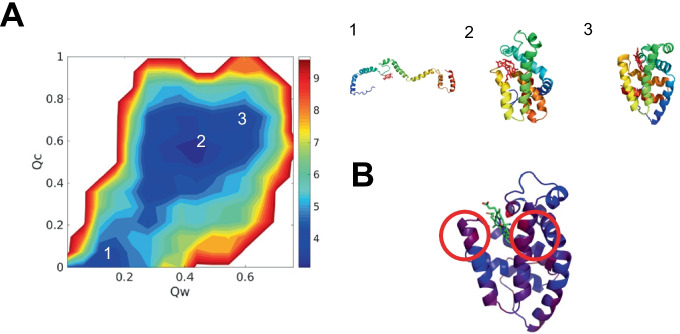
The free energy profile of 1F5O (hemoglobin) at temperature 300K. **A** the 2D free energy surface is plotted using the structural quality of protein $Q_{w}$ and the accuracy of protein–ligand position $Q_{c}$ as the two dimensions. The *Q* *w* is used to evaluate the similarity between simulated protein structure and experimentally determined protein structure. The $Q_{c}$ is used to measure the similarity between simulated protein-heme b pocket and crystal protein-heme b pocket. Represented structures are shown at the right panel, colored by a rainbow spectrum from red (N terminus) to blue (C terminus). **B** The best predicted structure is colored by RMSD, aligned to crystal structure from blue to red, from high RMSD values to low RMSD values. The regions of low RMSD values are circled

To better understand how the presence of heme b improves protein predicted structures, we projected the free energy landscape using the structural quality of the protein $Q_{w}$ and the accuracy of heme b ligation $Q_{c}$ as two dimensions. $Q_{w}$ has been widely used to measure the structural similarity with crystal structures while $Q_{c}$ evaluates the accuracy of heme b ligation.$Q_{c}$ varies from 0 to 1. Higher $Q_{c}$ structures position the heme more accurately in its native binding pocket. The free energy profile exhibits downhill characteristics, suggesting that heme b spontaneously moves to the native pocket during protein folding. Three representative structures are shown in the right panel in Fig. 2. The unfolded structure is shown in Fig. 2(A1), showing that heme b searches to bind with the protein. The folded structure is shown in Fig. 2(A3), where heme b has arrived at the native pocket. We see that heme b can find the binding pocket during the folding process. Another representative structure is shown in Fig. 2(A2), where heme b has reached the native pocket of the partially folded protein. Heme b forms contacts with the native pocket before the protein becomes completely folded, suggesting that heme b acts as a nucleation site for protein folding [42]. We have colored the best predicted structure in our simulation by RMSD values from blue to red, from high RMSD to low in Fig. 2(B). The residues around the heme b have low RMSD, indicating that heme b makes the pocket more similar to the crystal structure more strongly than other more distant residues. Heme b incorporation thus enhances the structural quality of the pocket geometry, and improves the structural quality of the protein structure prediction.

### Heme b finds native pocket driven by coordinated covalent bond

**Fig. 3:**
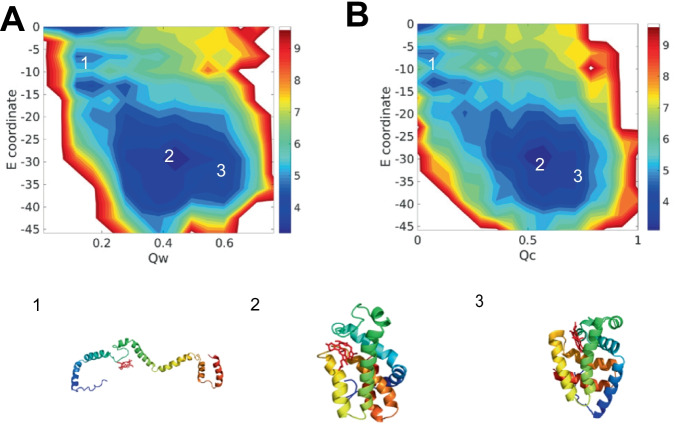
The free energy profile of 1F5O (hemoglobin) at temperature 300K. **A** the 2D free-energy surface is plotted using the accuracy of protein structure $Q_{w}$ and coordinated covalent bond energy as the two dimensions. **B** the 2D free-energy surface is plotted using the accuracy of protein–ligand position $Q_{c}$ and coordinated covalent bond energy as the two dimensions. Represented structures are shown at the bottom, colored by a rainbow spectrum from red (N terminus) to blue (C terminus)

The interactions between heme b and the surrounding protein are knowledge-based and transferable, the values being derived from experimental values. To better understand the role of the different intermolecular forces during protein folding, we computed several different free energy profiles using structural quality measures appropriate on different levels ($Q_{w}$, $Q_{c}$) and used serveral different intermolecular interaction energies as two dimensions. In our model, heme b has two types of functional groups, the iron group and two carboxyl groups. Iron, the active center of heme b, forms coordinate covalent bonds with histidines, methionines, or small molecules such as water and oxygen. To understand the role of forming the coordinated covalent bond in the folding process, we projected the 2D free energy profile using structural quality ($Q_{w}$, $Q_{c}$) and coordinated covalent bond energy, shown in Fig. 3. Both free energy landscapes are downhill, indicating the coordinated covalent bond is correlated to the protein folding and ligand searching process. Heme b reaches the native pocket both in the partially folded state and in the folded state from the last section. The slight difference between the coordinated covalent bond energy of the partially folded state and of the folded state shows that the coordinated covalent bond directly influences the heme b search for the binding pocket, and thus indirectly affects the folding process.

Besides the iron, the carboxyl group takes part as hydrogen bonds form with residues or water. We divided this interaction into three parts: hydrogen bonds formation with protein backbone, hydrogen bond formation with protein sidechains, hydrogen bond formation with water. The landscapes plotted between the structure quality measures and hydrogen bond formation energywith residues are shown in Fig. S3. The correlation between hydrogen bond formation with the residues and overall structural quality is weak, indicating the hydrogen bonds between the heme b and residues do not primarily help the heme b find its native pocket. But as shown in Fig. S2, the free energy landscapes for hydrogen bonds formation with water and structural quality are not downhill. This energy difference between the unfolded state and the folded state indicates that carboxyl groups of heme b don’t strongly prefer to interact with water during ligand searching.

We have also included the nonbonded terms in our model as the Van der Waals interaction and electrostatics. Because AWSEM is a coarsed-grained model, the Van der Waals interaction mostly plays a repulsive role between protein and heme b. To better understand the role of electrostatics, we also constructed the 2D free energy profile using structural quality and electrostatic energy in Fig. S1. The differences between the unfolded states, the partially folded state, and the folded states are obvious but are weaker than other interactions in magnitude, suggesting that electrostatics does encourage the folded process. Considering the magnitudes of each interaction, the heme b search for the binding pocket is dominated by forming coordinated covalent bonds between the iron and protein.

### Heme c incorporation enhances the accuracy of structure prediction

**Fig. 4:**
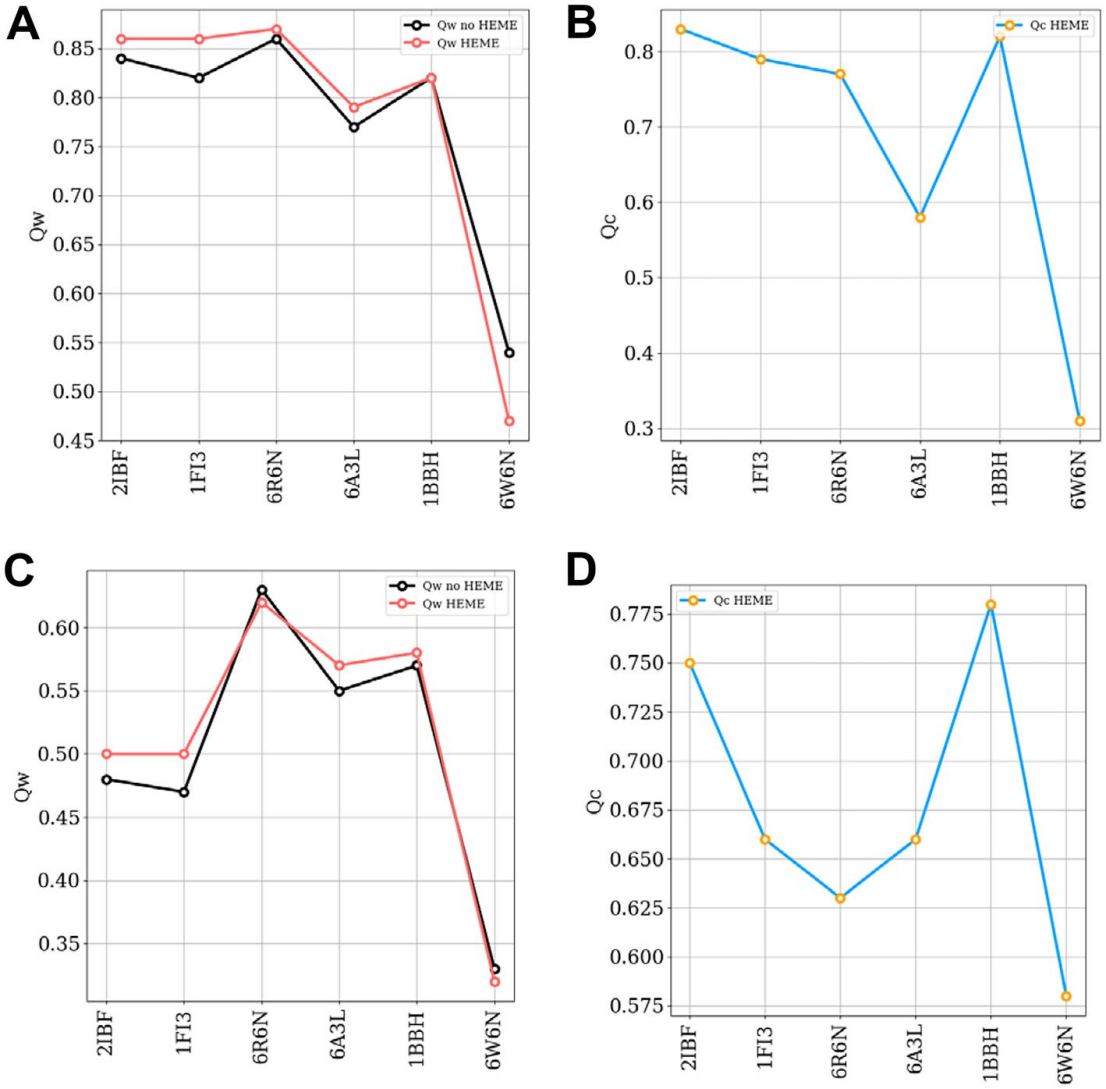
Summary of heme c protein predictions. **A** The $Q_{w}$ between best structures in predictions and experimental determined structures by different setups using single memory. Black: Apo form, Red: Holo form. **B** The $Q_{c}$ between best structures in holo form predictions using single memory and experimental determined structures. **C** The $Q_{w}$ between best structures in predictions and experimental determined structures by different setups using fragment memory. Black: Apo form, Red: Holo form. **D** The $Q_{c}$ between best structures in holo form predictions using fragment memory and experimental determined structures

In contrast to heme b, the vinyl groups of heme c form two carbon–sulfide covalent bonds with cysteines. Being restricted by the distance of two vinyl groups, the bound cysteines are often separated by two residues. We added these constraints to our model and summarized the heme c protein structure prediction results using a single memory in Fig. 4. We compared the maximum $Q_{w}$ values predicted by the apo form (Black) and the holo form (Red) simulations versus sequence length in Fig. 4(A) and plotted the $Q_{c}$ values of corresponding structures in Fig. 4(B). Without heme c, the predictions of protein by AWSEM are reasonably good, suggesting that protein can fold toward native state even without heme c. Most results of the holo form predictions nevertheless are improved compared to the apo form predictions. The slight improvement of the holo form predictions comes from the already accurate prediction of apo form that the $Q_{w}$ are above 0.77. But the predictions for one target (PDBID:6W6N) are worse than for the apo form when it is less similar to the crystal structure, suggesting that heme c improves the structural prediction only when the apo form prediction is accurate enough. Judging from the heme c position’s accuracy, we see it can find the native pocket when the predicted structure folded toward the native state. But when the apo form prediction is not accurate, the heme c can be misled to bind to a non-native pocket. We summarize the prediction results using fragment memories in Fig. 4. For the apo form, AWSEM using fragment memories performs worse than AWSEM using a single memory again as expected. Though all heme c’s can find their native pocket, the holo form predictions improve in quality slightly when the apo form’s prediction is accurate enough.

### Heme c can find native pocket during protein folding

**Fig. 5:**
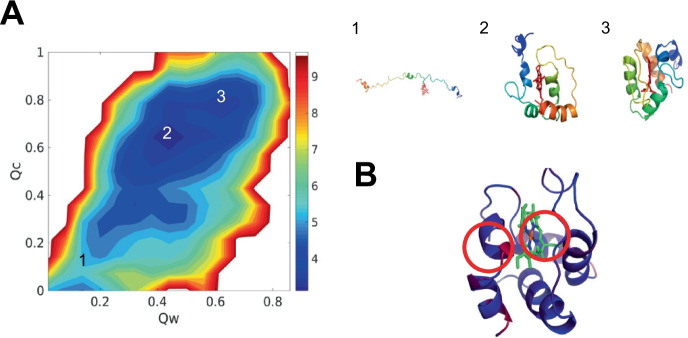
Grand canonical free energy profile of 1FI3 (cytochrome c) at temperature 300K. **A** the 2D free energy surface is plotted using the structural quality of protein $Q_{w}$ and the accuracy of protein–ligand position $Q_{c}$ as the two dimensions. The $Q_{w}$ is used to evaluate the similarity between simulated protein structure and experimentally determined protein structure. The $Q_{c}$ is used to measure the similarity between simulated protein-heme c pocket and crystal protein-heme c pocket. Represented structures are shown at the right panel, colored by a rainbow spectrum from red (N terminus)to blue (C terminus). **B** The best predicted structure is colored by RMSD, aligned to crystal structure from blue to red, from high RMSD values to low RMSD values. The regions of low RMSD values are circled

To better understand how the introduction of heme c improves protein structure predictions, we projected the free energy landscape using the structural quality measure for the protein $Q_{w}$ and the accuracy of heme c ligation quality measure $Q_{c}$ as two dimensions. The free energy profile is downhill, suggesting that heme c finds the native pocket as the protein folds. Three represented structures are shown in the right panel in Fig. 5. Heme c searches along with the unfolded structure (Fig. 5(A).1) and arrives at the native pocket in the folded structure (Fig. 5(A).3). Heme c reaches the native pocket even in the partially folded protein: the native pocket with heme c forms before the protein folds. These results suggest that heme c acts as a nucleation site for protein folding. We also have colored the best folded structure in our simulations by RMSD values in Fig. 5(B). The residues around heme c have low RMSD, indicating that heme c influences the pocket morethan the more distant residues. Heme c incorporation improves the structural quality of the pocket but does not greatly affect the long-range interactions.

### Heme c finds its native pocket driven by thioester and coordinated covalent bonds

**Fig. 6:**
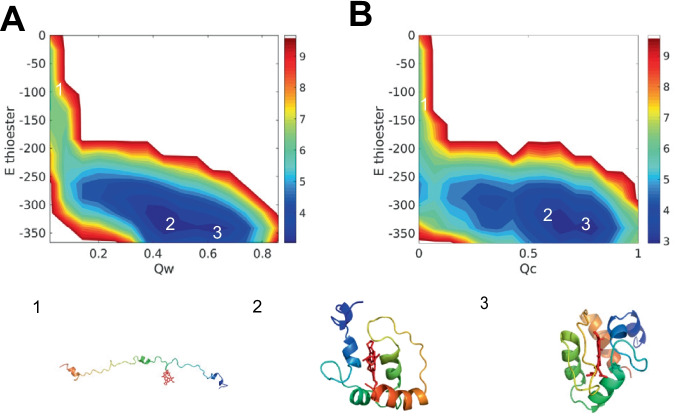
The free energy profile of 1FI3 (cytochrome c) at temperature 300K. **A** the 2D free energy surface is plotted using the accuracy of protein structure $Q_{w}$ and thioester covalent bond energy as the two dimensions. **B** the 2D free-energy surface is plotted using the accuracy of protein–ligand position $Q_{c}$ and thioester covalent bond energy as the two dimensions. The represented structures are shown at the bottom, colored by a rainbow spectrum from red (N terminus) to blue (C terminus)

Here we survey the roles of the different intermolecular forces in the energy landscape by computing several different free energy profiles that use structural quality measures and the different intermolecular interaction energies as dimensions. The two vinyl groups of the heme c form thioester covalent bonds with the protein. To better understand the function of thioester covalent bonds, we constructed the 2D free energy profile using structural quality ($Q_{w}$, $Q_{c}$) and thioester covalent bond energy, shown in Fig. 6. Two landscapes exhibit downhill characteristics, indicating the thioester covalent bonds help the protein fold and help the heme c search for the binding pocket during the folding process. The significant difference of thioester covalent bond energy between the unfolded state and the partially folded state and the smaller difference of thioester bond energy between the partially-folded state and the folded state shows that the thioester covalent bond helps heme c form contacts with pocket before the protein folds. Therefore, the thioester covalent bonds directly influence the interactions between heme c and native pocket.

Considering the active center of iron, we projected the 2D free energy profile using structural quality measures ($Q_{w}$, $Q_{c}$) and coordinated covalent bond energy. As in the heme b case, the downhill landscapes indicate that the heme c binding process is funneled using the coordinated covalent bond. The slight differences in heme c ligation and significant difference in protein structure between partially-folded state and folded state mean that the coordinated covalent bond helps the heme c form contacts with the native pocket and thereby affects the landscape of the whole protein.

Examining interactions with the carboxyl groups, we computed several landscapes using structural quality ($Q_{w}$, $Q_{c}$) and the varing different hydrophilic interactions as dimensions. The landscapes for hydrogen bonds formation with residues and the landscapes for hydrogen bonds formation with water are not downhill, suggesting that these hydrogen bonds do not help heme c find the native pocket during protein folding.

To understand the role of electrostatics, we projected the 2D free energy profile using electrostatic energy and structural quality. The electrostatics can distinguish unfolded states, partially folded states and the folded states, suggesting that electrostatics help stabilize the folding funnel. We see that the heme c binding process is driven by the formation of thioester covalent bonds between vinyl groups and cysteines and the formation of the coordinate covalent bonds between iron and the protein residues.

## Discussion

### Heme c stabilizes the folded state of heme proteins

The hybrid transferable AWSEM-heme model provides significant improvement over the pure AWSEM model in moderate-resolution structure prediction. Holo form prediction, however only, performs better than apo form prediction when the apo form predicted structures are already sufficiently accurate. In heme b proteins, the binding process is driven by forming the coordinate covalent bond, and is also stabilized by electrostatics. In heme c proteins, the binding is driven by the thioester covalent bond and the coordinated covalent bond, also stabilized by electrostatics. Hydrogen bond formation between carboxyl groups and residues does not show any specific preference during the heme searching process.

**Fig. 7:**
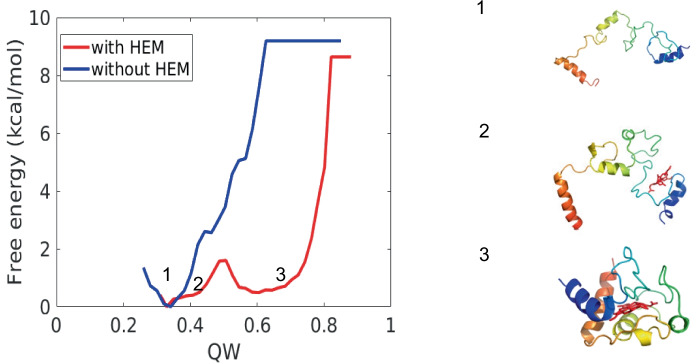
The diagram shows the effect of heme c on the folding process. Free energy profiles are plotted as a function of reaction coordinated $Q_{w}$ of protein using different setups. Blue: without heme c, Red: with heme c

Heme b and heme c also act as nucleation sites for protein folding. Both heme b and heme c form contacts with native pocket before the protein folds completely. To better understand the effects of heme on protein folding, umbrella-sampling simulations of the apo and holo form using the $Q_{w}$ of the determined structure (PDB ID: 1HRC) as the biasing coordinate were carried out to construct free-energy profiles. Apo form simulations were carried out using the traditional AWSEM model, and holo form simulations were carried out using the hybrid AWSEM-heme model. As shown in Fig. 7, at 300K (the folding temperature of holo form), only the unfolded state whose $Q_{w}$ around 0.2 is stable in the apo form. In the holo form, not only is the unfolded state $Q_{w}$ at about 0.2 stable, but also the folded state $Q_{w}$ around 0.6 is stabilized by the heme c binding. These suggest that the interactions between the heme c and sidechains of the protein significantly promote protein stability and folding, which agrees with Weinkam et al.’s work [42]. Representative structures from these basins are also shown in Fig. 7. The $Q_{w}$ of unfolded states of the apo and holo form are around 0.3, the unfolded state of holo form is more compact than the unfolded apo form, suggesting that heme c acts as a nucleation site to promote protein folding.

In this paper, we have developed a transferable and knowledge-based AWSEM-heme model, which successfully improves structure prediction for these important molecules. Also, we surveyed the role of the interactions between heme and protein during protein folding. Heme c directly influences the structure and search for the native pocket, therefore indirectly affecting the protein’s architecture and function. We plan to investigate further the heme’s effects on the dynamics of hemoglobin, myoglobin, and neuroglobin using this tool in the future.

There are still several limitations to our model. For example, we have not optimized the charge distribution of the heme and the protein, that limits us from studying the electron transfer process. We did not build a suitable model of a small molecule such as O_2_, which restricts us from learning the oxidation process. Thus, the accuracy of our model is not enough to study the pathway without the second effect of iron ions. We will continue to improve this model and apply it to study more biological processes.

## Supplementary information

Below is the link to the electronic supplementary material.

Supplementary file1 (DOCX 2220 KB)

## Data Availability

All the data for this paper are available at https://github.com/chemlover/heme_prediction.

## Code availability

The simulation and analysis codes are available online at https://github.com/chemlover/heme_prediction.
