# Supplementary material for: Exploring the folding energy landscapes of heme proteins using a hybrid AWSEM-heme model
Source: J Biol Phys. 2022 Jan 9;48(1):37–53. doi: 10.1007/s10867-021-09596-3 (PMC8866609; doi:10.1007/s10867-021-09596-3)
Supplement: Supplementary file 1 — Supplementary file1 (DOCX 2220 KB) [file 10867_2021_9596_MOESM1_ESM.pdf]

Supporting information for exploring the folding energy landscapes of heme proteins using a hybrid AWSEM-heme model

Xun Chen,[†,‡,¶] Wei Lu,[†,§,¶] Min-Yeh Tsai,[‖] and Peter G. Wolynes[*,†,‡,⊥]

†*Center for Theoretical Biological Physics*

‡*Department of Chemistry, Rice University*

¶*Equal Contribution*

§*Department of Physics, Rice University*

‖*Department of Chemistry, Tamkang University, New Taipei City 25137, Taiwan (R.O.C.)*

⊥*Department of Biosciences, Rice University*

E-mail: pwolynes@rice.edu

Phone: (713)348-4101

1 Heme b incorporation enhances the accuracy of structure prediction

Table 1: The Summary of Comparison between apo form and holo form using singe memory for heme b proteins

Target Information		structural quality		
Target Name	Length	Qw of apo form	Qw of holo form	Qc of holo form
6ZMX	142	0.76	0.75	0.75
1A01	145	0.66	0.68	0.55
1W92	150	0.76	0.77	0.8
1F5O	153	0.75	0.76	0.59
5XKW	153	0.63	0.73	0.88
5YCI	158	0.76	0.76	0.71

Table 2: The summary of comparison between apo form and holo form using fragment memory for heme b proteins

Target Information		structural quality		
Target Name	Length	Qw of apo form	Qw of holo form	Qc of holo form
6ZMX	142	0.71	0.75	0.8
1A01	145	0.78	0.78	0.95
1W92	150	0.43	0.45	0.75
1F5O	153	0.44	0.39	0.27
5XKW	153	0.42	0.51	0.72
5YCI	158	0.5	0.53	0.11

2 The contacts formed by heme b and native pocket are stabilized by electostatic

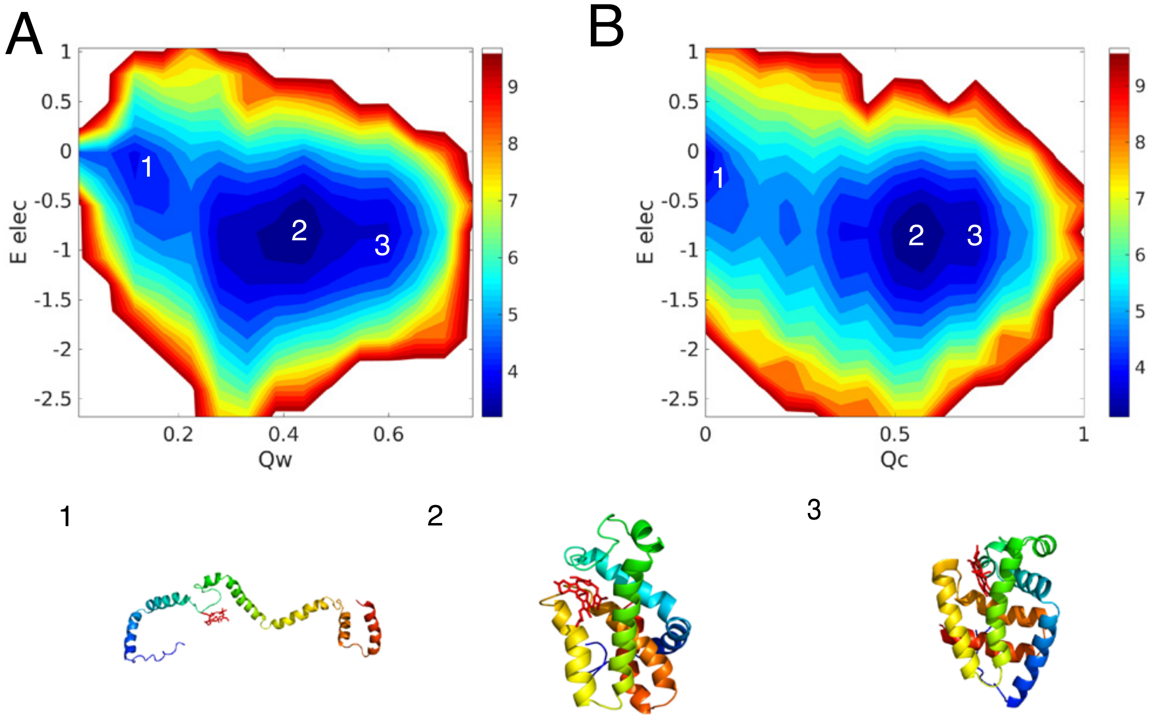

Figure 1: The free energy profile of 1F5O (hemoglobin) at temperature 300K. A) the 2D free-energy surface is plotted using the accuracy of protein structure Q_w and electrostatics energy as the two dimensions. B) the 2D free-energy surface is plotted using the accuracy of protein-ligand position Q_c and electrostatics energy as the two dimensions. Represented structure are shown at the bottom, colored by a rainbow spectrum from red (N terminus)to blue (C terminus)

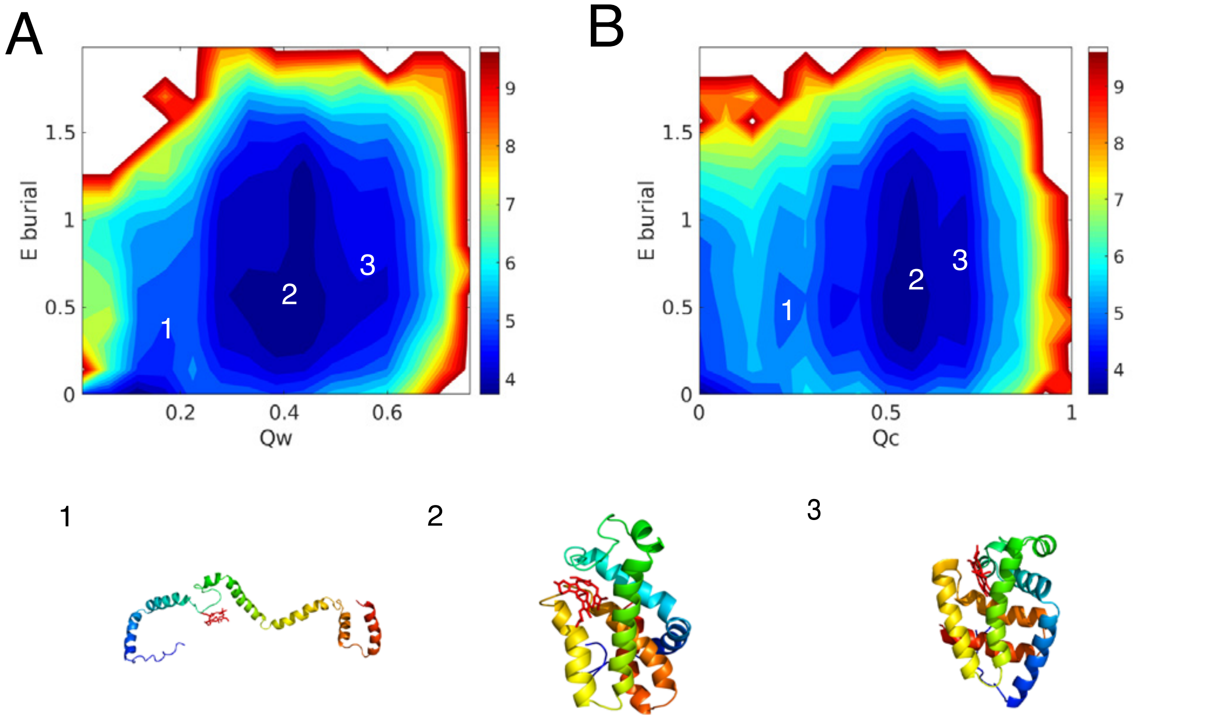

Figure 2: The free energy profile of 1F5O (hemoglobin) at temperature 300K. A) the 2D free-energy surface is plotted using the accuracy of protein structure Q_w and burial energy as the two dimensions. B) the 2D free-energy surface is plotted using the accuracy of protein-ligand position Q_c and burial energy as the two dimensions. Represented structure are shown at the bottom, colored by a rainbow spectrum from red (N terminus)to blue (C terminus)

3 Hydogen bonds formed with specific residues can not distingustish different binding states in heme b proteins

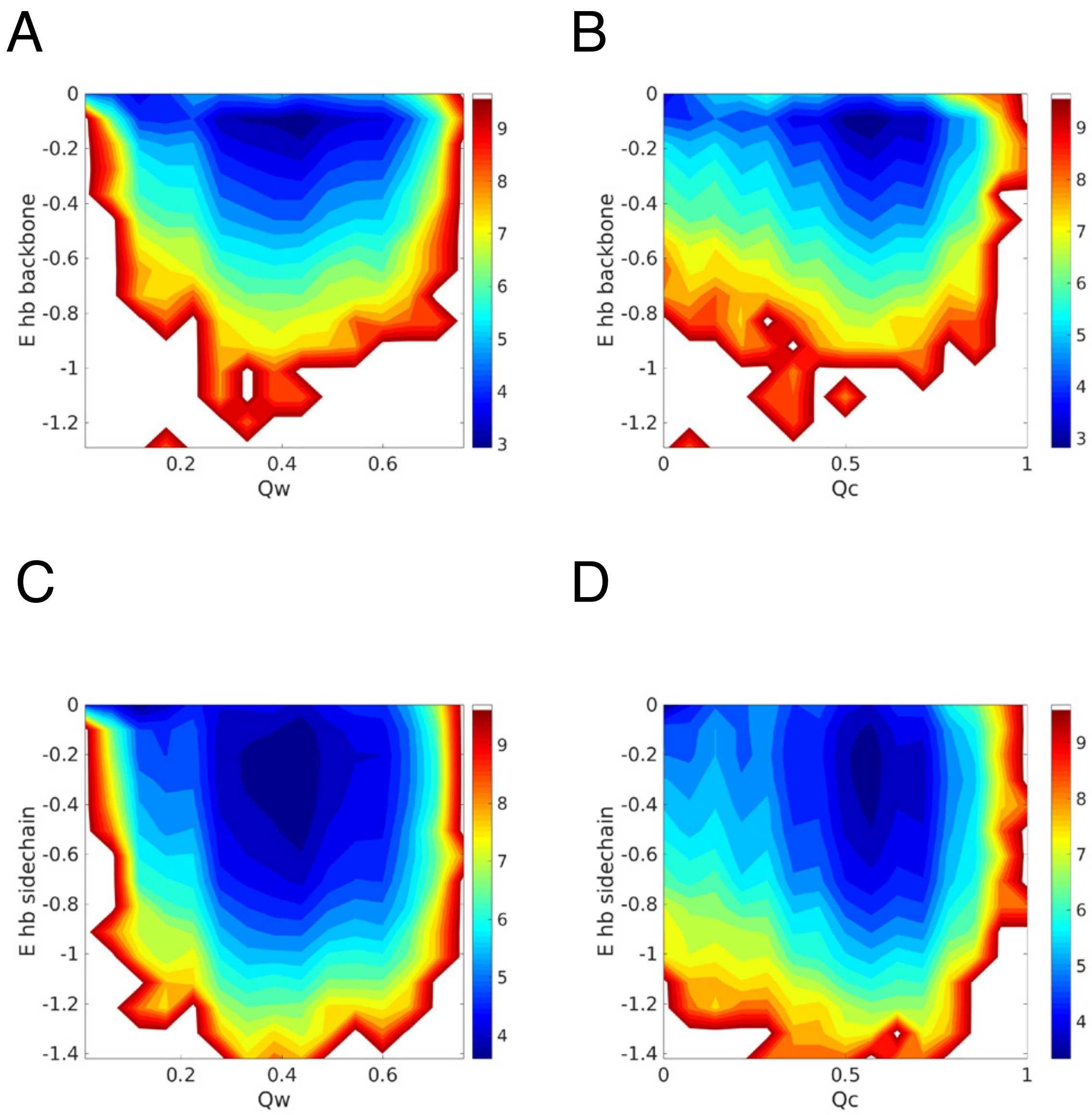

Figure 3: A) 2d free energy landscape projected on hydrogen bondsformation with backbone and Qw.B) 2d free energy landscape projected on hydrogen bonds formation with sidechain and Qc. C) 2d free energy landscape projected on hydogen bonds formation with backbone and Qc,D) 2d free energy landscape projected on hydrogen bonds formation with sidechain Qc

4 Heme c incorporation enhances the accuracy of structure prediction

Table 3: The summary of comparison between apo form and holo form using singe memory for heme c proteins

Target Information		structural quality		
Target Name	Length	Qw of apo form	Qw of holo form	Qc of holo form
2IBF	82	0.84	0.86	0.83
1FI3	86	0.82	0.86	0.79
6R6N	115	0.84	0.87	0.77
6A3L	133	0.77	0.79	0.66
1BBH	135	0.82	0.82	0.82
6W6N	162	0.54	0.47	0.31

Table 4: The summary of comparison between apo form and holo form using fragment memory for heme c proteins

Target Information		structural quality		
Target Name	Length	Qw of apo form	Qw of holo form	Qc of holo form
2IBF	82	0.48	0.5	0.75
1FI3	86	0.47	0.5	0.66
6R6N	115	0.63	0.62	0.63
6A3L	133	0.55	0.57	0.66
1BBH	135	0.57	0.58	0.78
6W6N	162	0.33	0.32	0.58

5 Heme c finds its native pocket driven by thioeste andr coordinated covalent bonds

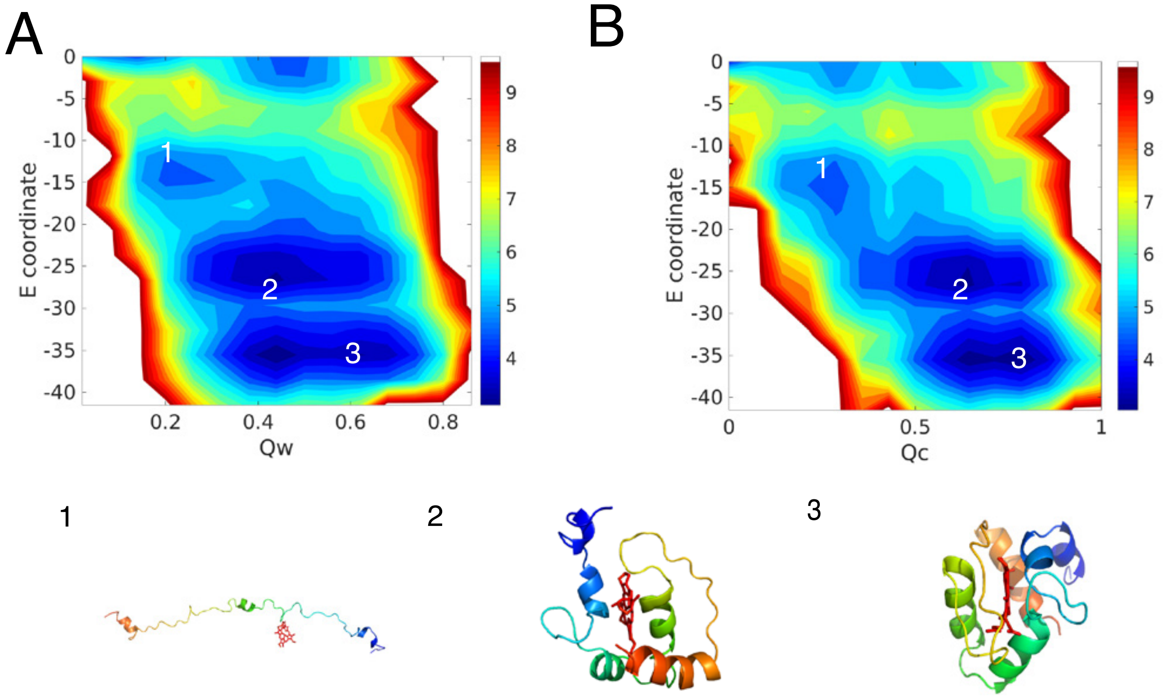

Figure 4: The free energy profile of 1FI3 (cytochrome c) at temperature 300K. A) the 2D free-energy surface is plotted using the accuracy of protein structure Q_w and coordinated covalend bond energy as the two dimensions. B) the 2D free-energy surface is plotted using the accuracy of protein-ligand position Q_c and coordinated covalend bond energy as the two dimensions. Represented structure are shown at the bottom, colored by a rainbow spectrum from red (N terminus)to blue (C terminus)

6 The contacts formed by heme c and native pocket are stabilized by electostatic

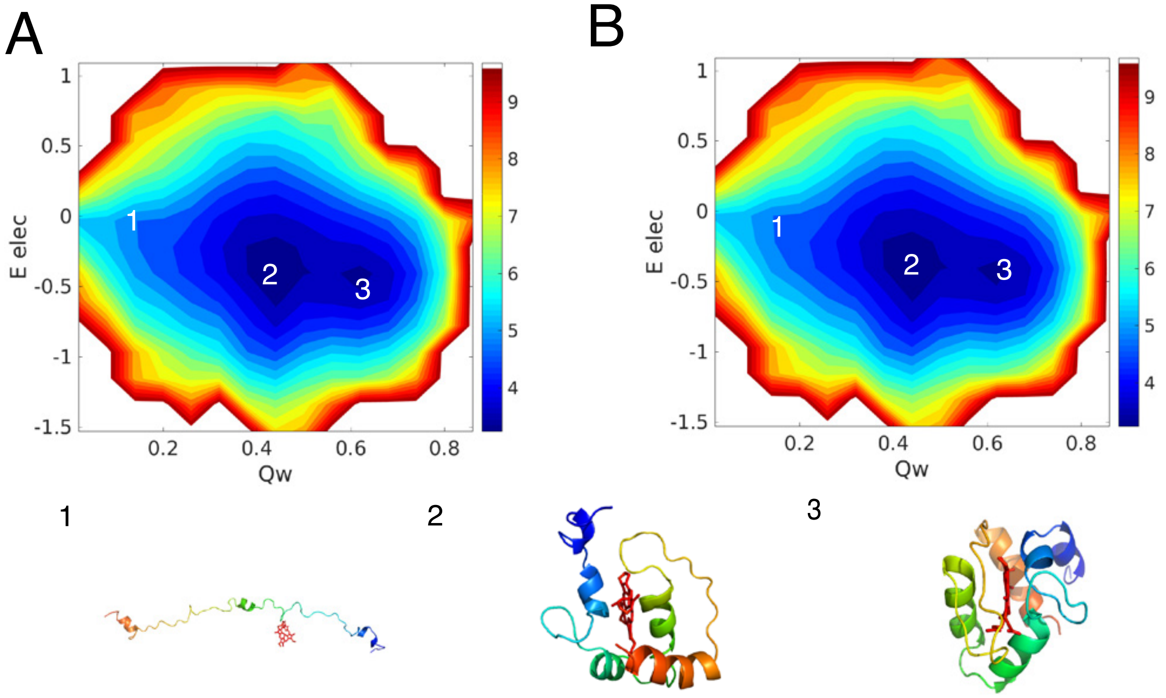

Figure 5: The free energy profile of 1FI3 (cytochrome c) at temperature 300K. A) the 2D free-energy surface is plotted using the accuracy of protein structure Q_w and electrostatics energy as the two dimensions. B) the 2D free-energy surface is plotted using the accuracy of protein-ligand position Q_c and electrostatics energy as the two dimensions. Represented structure are shown at the bottom, colored by a rainbow spectrum from red (N terminus)to blue (C terminus)

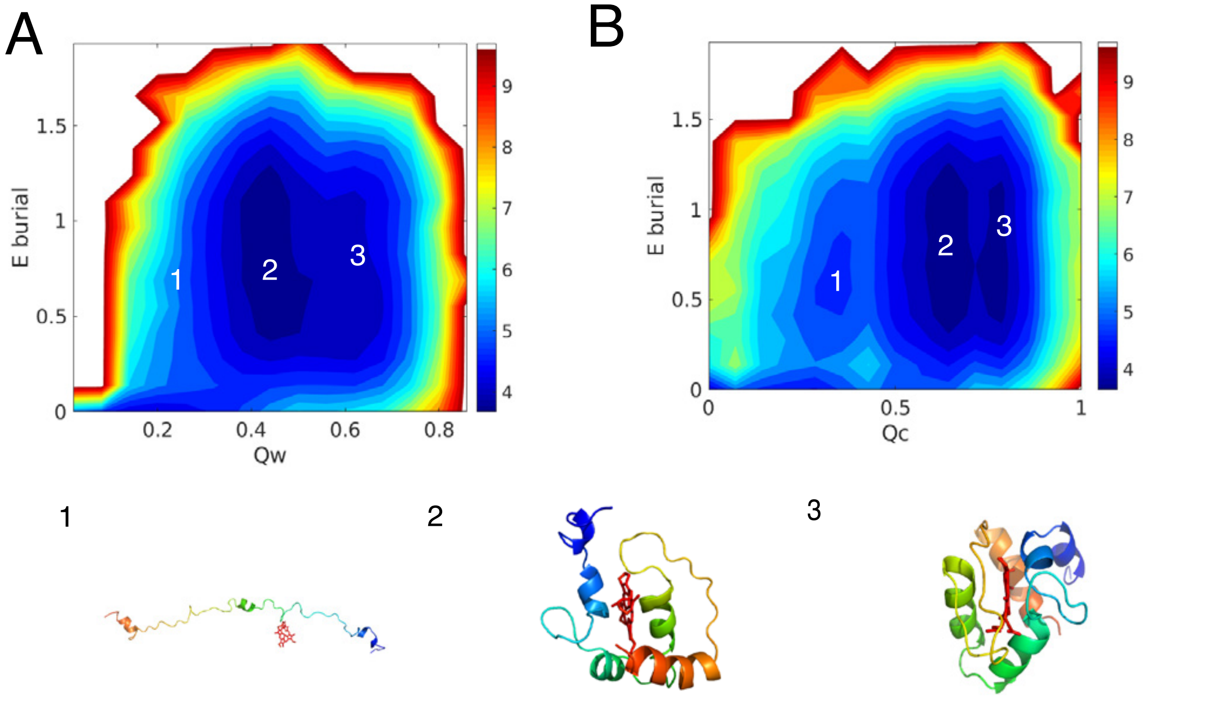

Figure 6: Thefree energy profile of 1FI3 (cytochrome c) at temperature 300K. A) the 2D free-energy surface is plotted using the accuracy of protein structure Q_w and burial energy as the two dimensions. B) the 2D free-energy surface is plotted using the accuracy of protein-ligand position Q_c and burial energy as the two dimensions. Represented structure are shown at the bottom, colored by a rainbow spectrum from red (N terminus)to blue (C terminus)

7 Hydogen bonds formed with specific residues can not distingustish different binding states in heme c protein

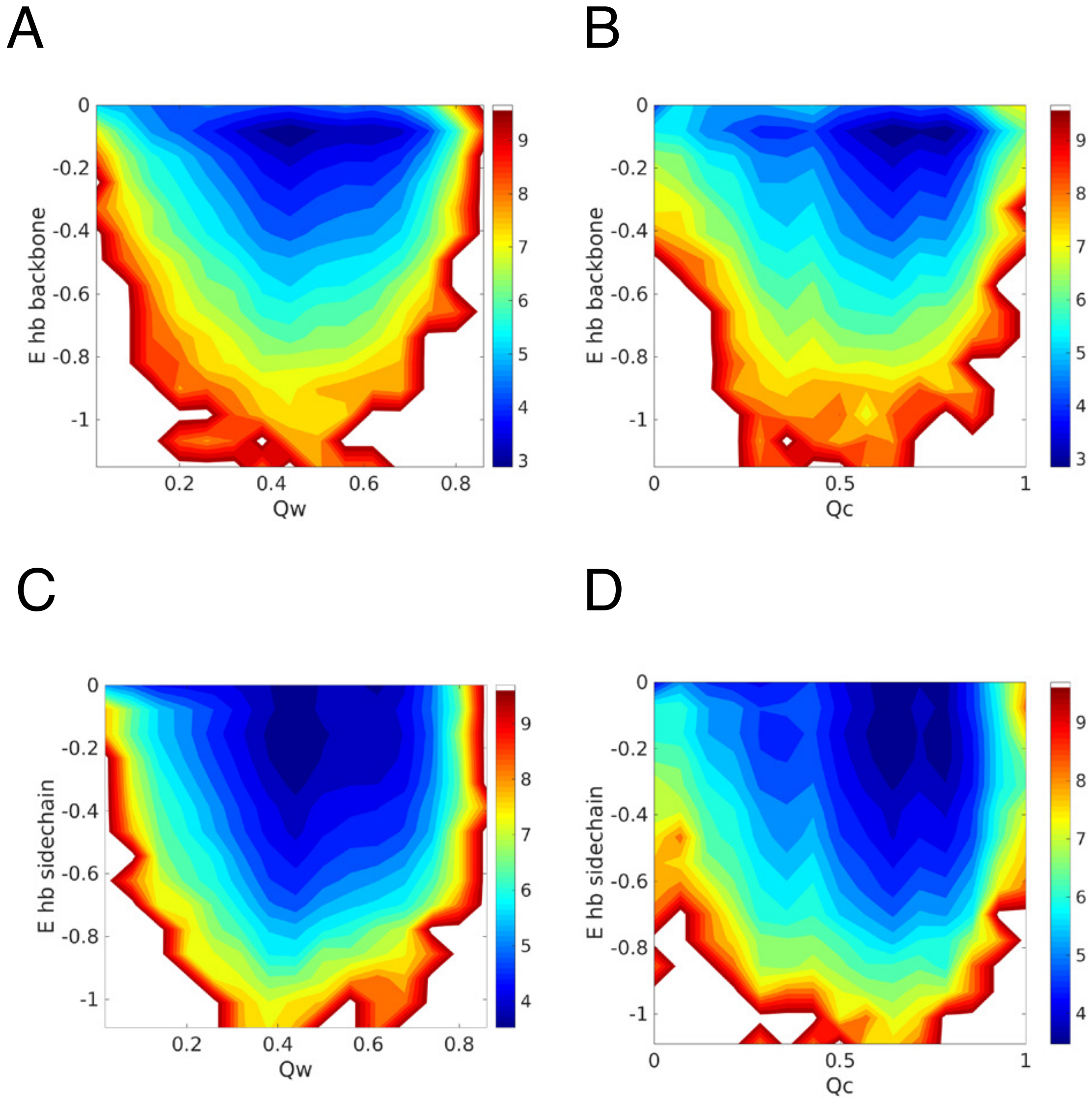

Figure 7: A) 2d free energy landscape projected on hydrogen bondsformation with backbone and Qw.B) 2d free energy landscape projected on hydrogen bonds formation with sidechain and Qc. C) 2d free energy landscape projected on hydogen bonds formation with backbone and Qc,D) 2d free energy landscape projected on hydrogen bonds formation with sidechain Qc

8 Parameters in Methods

Table 5: Paramters used in $V_{hb-sidechain}$

Parameter	Value	Units
λ_{Fe-H}	14	kcal/mol
λ_{Fe-M}	7	kcal/mol
λ_{Fe-Y}	7	kcal/mol
λ_{Fe-F}	7	kcal/mol
λ_{Fe-C}	1.4	kcal/mol
r_{Fe-cc}	0.57	nm
θ_{Fe-cc}	1.57	Rad

Table 6: Paramters used between heme and protein

Parameter	Value	Units
λ_{elec}	4.15	kcal/mol
$\lambda_{hb-backbone}$	1.9	kcal/mol
λ_{burial}	-1.9	kcal/mol
λ_{excl}	5	kcal/mol
$\lambda_{thioester}$	175	kcal/mol
$l_{screening}$	1	nm
$k_{screening}$	1	kcal/mol
r_{HB}	0.16	nm
θ_{HB}	1.57	Rad
r_{burial}	0.6	nm
r_{excl}	0.3	nm
$r_{thioester}$	0.28	nm

Table 7: Paramters used in $V_{hb-sidechain}$

Parameter	Value	Units
$\lambda_{Y,HB}$	2.28	kcal/mol
$\lambda_{W,HB}$	0.76	kcal/mol
$\lambda_{K,HB}$	1.9	kcal/mol
$\lambda_{R,HB}$	1.52	kcal/mol
$\lambda_{H,HB}$	1.52	kcal/mol
$\lambda_{D,HB}$	1.33	kcal/mol
$\lambda_{E,HB}$	1.33	kcal/mol
$\lambda_{N,HB}$	2.28	kcal/mol
$\lambda_{Q,HB}$	2.28	kcal/mol
$\lambda_{C,HB}$	1.14	kcal/mol
$\lambda_{S,HB}$	2.28	kcal/mol
$\lambda_{T,HB}$	2.28	kcal/mol
$r_{Y,HB}$	0.96	nm
$r_{W,HB}$	0.56	nm
$r_{K,HB}$	0.96	nm
$r_{R,HB}$	1.06	nm
$r_{H,HB}$	0.76	nm
$r_{D,HB}$	0.76	nm
$r_{E,HB}$	0.76	nm
$r_{N,HB}$	0.76	nm
$r_{Q,HB}$	0.96	nm
$r_{C,HB}$	0.46	nm
$r_{S,HB}$	0.46	nm
$r_{T,HB}$	0.46	nm
